# Dietary Patterns, Hepatic Fat Fraction, and the Role of Genotype

**DOI:** 10.3390/nu18071087

**Published:** 2026-03-28

**Authors:** Kyle Salmon, Catherine C. Cohen, Leslie Lange, Dana Dabelea, Wei Perng

**Affiliations:** 1 Lifecourse Epidemiology of Adiposity and Diabetes (LEAD) Center, University of Colorado Anschutz Medical Campus, Aurora, CO 80045, USA; kyle.salmon@cunaschutz.edu (K.S.); catherine.cohen@cuanschutz.edu (C.C.C.); dana.dabelea@cuanschutz.edu (D.D.); 2 Department of Pediatrics, University of Colorado School of Medicine, Aurora, CO 80045, USA; 3 Division of Biomedical Informatics and Personalized Medicine, Department of Medicine, University of Colorado Anschutz Medical Campus, Aurora, CO 80045, USA; leslie.lange@cuanschutz.edu; 4 Department of Epidemiology, School of Public Health, University of Colorado Anschutz Medical Campus, Aurora, CO 80045, USA; 5 Department of Nutritional Sciences, University of Michigan School of Public Health, Ann Arbor, MI 48109, USA

**Keywords:** metabolic dysfunction-associated steatotic liver disease (MASLD), hepatic fat fraction (HFF), diet, adolescents, epidemiology

## Abstract

**Background/Objectives:** We aimed to identify eating habits associated with hepatic fat fraction (HFF) and assess effect modification by an established genetic variant for fatty liver disease, *PNPLA3 rs738409*, among 381 general-risk adolescents. **Methods**: Dietary intake was assessed using the Block Kids Food Frequency Questionnaire and HFF was measured via magnetic resonance imaging (MRI) at age ~16 years. We first characterized naturally occurring dietary patterns using principal component analysis followed by reduced-rank regression with HFF as the response variable to identify a dietary pattern that is both relevant to the population and associated with HFF. Next, we investigated associations of the dietary pattern with HFF using linear regression models that accounted for maternal gestational diabetes, education, and prenatal smoking and child sex, age, Tanner stage, and BMI. Finally, we tested for a dietary pattern and *PNPLA3 rs738409* interaction and stratified by genotype if *P*-interaction < 0.05. **Results**: The participants were 16.7 ± 1.2 years (range: 12.6–19.6 years). Half were female (50.4%) and 52.0% identified as non-Hispanic White. The dietary pattern of interest was composed of vegetables, fruit, nuts and seeds, oatmeal, sports bars, crackers and sandwiches, and beef, and was inversely associated with HFF (−0.48 [95% CI: −0.81, −0.16]). Stratified analyses revealed the strongest inverse association observed between the diet pattern score and HFF in the high-risk-variant (GG) group (−2.19 [−4.35, −0.03]), followed by the intermediate-risk (CG) group (−0.43 [−0.77, −0.10]), but not the low-risk (CC) group (−0.32 [−0.77, 0.13]). **Conclusions**: A diet high in vegetables, fruit, nuts and seeds, oatmeal, sports bars, crackers and sandwiches, and beef—potentially capturing an active, on-the-go lifestyle—is associated with lower HFF during adolescence, especially among individuals at genetic risk.

## 1. Introduction

Hepatic fat fraction (HFF) is a measurement of how much fat is present in the liver. HFF is commonly used to evaluate the risk of fatty liver disease, ranging from mild steatosis to advanced metabolic dysfunction-associated steatotic liver disease (MASLD), the most common chronic liver disease worldwide [1]. The rise of the childhood obesity epidemic has contributed to an increase in MASLD prevalence among children and adolescents, now affecting approximately 10% of the general pediatric population and up to 35% of youth with obesity [2].

The causes of MASLD are complex [3] and likely involve gene-by-environment interactions. Specifically, the GG variant of the *PNPLA3 rs738409* genotype has been linked to liver fat accumulation, hepatic inflammation, steatohepatitis, and fibrosis and cirrhosis [4]. Given the absence of approved pharmacological treatments for MASLD in pediatric populations, lifestyle modifications remain the primary strategy for managing youth-onset MASLD [5,6].

Several studies have linked dietary habits to risk of MASLD. In adults, a recent systematic review highlights that intake of refined carbohydrates and unhealthy fats are consistently associated with risk of MASLD [7]. Conversely, both observational studies and clinical trials have demonstrated that adherence to a Mediterranean-style or “prudent” diet characterized by high consumption of polyunsaturated fats, fresh fruits, vegetables, and dietary fiber is protective against MASLD [8]. However, a limitation of the current literature is the predominant use of unsupervised approaches to characterize dietary patterns and/or derivation of a priori diet indices (e.g., Healthy Eating Index, Mediterranean Diet Score) that have varying degrees of relevance to the study population.

In the Exploring Perinatal Outcomes among CHildren (EPOCH) cohort, a cohort of general-risk adolescents in Denver, CO, we identified two dietary patterns prospectively and cross-sectionally associated with hepatic fat fraction (HFF) using principal component analysis (PCA), an unsupervised dimension-reduction approach that characterizes data-driven, naturally occurring dietary patterns. Adherence to a “prudent” dietary pattern, characterized by high fruit and vegetable intake, was associated with lower HFF among all youth. In contrast, adherence to a Western dietary pattern was associated with higher HFF, but this association was observed only among non-Hispanic White youth [9].

We now seek to build upon our prior work by using both PCA and reduced-rank regression (RRR), a supervised dimension-reduction technique, to identify dietary patterns that are both contextually relevant and associated with HFF and, accordingly, fatty liver disease. In addition, given the known gene-by-environment interactions involved in shaping risk of MASLD, we assessed the *PNPLA3 rs738409* genotype as an effect modifier. We hypothesized that the integrated PCA-RRR approach would identify a dietary pattern that is both culturally relevant and associated with HFF and that associations of the dietary pattern with HFF and MASLD would differ by *PNPLA3 rs738409* genotype.

## 2. Materials and Methods

### 2.1. Study Population

Study participants were from the Exploring Perinatal Outcomes among CHildren (EPOCH) study, a historical prospective cohort that sought to understand the long-term consequences and mechanisms linking in utero overnutrition to the development of metabolic risk across childhood and adolescence. In total, 604 children (N = 99 children exposed to maternal diabetes; N = 505 a random sample of unexposed youth) aged 6–13 years and their mothers were enrolled in the EPOCH study between 2006 and 2010. The offspring returned for clinical research visits at ages ~10 (range: 6.0–13.9 years) and ~16 years (range: 12.6 to 19.6 years), during which we collected data on sociodemographic characteristics, anthropometry, and lifestyle behaviors, and collected fasting blood. At the adolescent visit, we also conducted magnetic resonance imaging (MRI) scans to assess HFF.

This paper focused on data collected at the mid-to-late adolescent visit when HFF was measured. Of the 417 participants who attended the adolescent research visit, we excluded 18 participants due to missing data on HFF and 18 participants without data on dietary intake, yielding an analytic sample of 381 youth. The study protocol was approved by the Colorado Multiple Institutional Review Board. Informed consent was obtained from mothers and written assent was obtained from their offspring.

### 2.2. Dietary Assessment (Exposure)

The Block Kids Food Frequency Questionnaire (FFQ) [10] assesses how often 83 different foods were consumed over the past week, with response options to indicate how often each food or beverage was consumed ranging from “once a day” to “every day.”

Subsequently, the 83 food items were consolidated into 42 food groups based on their nutritional properties [9]. Total daily energy intake was estimated using the United States Department of Agriculture Food Composition Database [11] and each food group was energy-adjusted using the residuals method [9,12]. We used this data to characterize dietary patterns of interest, described in the Section 3.

### 2.3. Assessment of Hepatic Fat Fraction (Outcome)

Hepatic imaging was performed at the adolescent study visit via MRI, using a modification of the Dixon method [13,14]. HFF was calculated from the mean pixel signal intensity data for each flip angle acquisition. In the analysis, we prioritized assessing HFF continuously, due to the small percentage of participants with elevated liver fat consistent with MASLD (7.6% with HFF ≥ 5%).

### 2.4. Genotyping (Potenital Effect Modifier)

Standard genotyping procedures were applied as previously detailed by Stanislawski et al., who examined genetic determinates of hepatic fat as a continuous measure within the same cohort [15]. In brief, DNA was isolated from stored peripheral venous blood using the QIAamp kit (Qiagen, Germantown, MD, USA) [16]. DNA quantification and purity were assessed using a NanoDrop spectrophotometer and a Qubit fluorometer (Thermo Scientific, Waltham, MA, USA).

Genotyping was completed in 2 batches: the first batch utilized the Illumina Infinium Omni2.5-8 v1.1 (Illumina, Inc., San Diego, CA, USA) BeadChip for 226 samples, and the second batch used the Illumina Multi-Ethnic Global Array v1.0 (Illumina, Inc.) for 130 samples. QA/QC and filtering were conducted using PLINK 1.9 (available at www.cog-genomics.org/plink/1.9 (accessed on 25 December 2025)), following protocols described previously [17]. Participants with ≥5% missing genotypes and single nucleotide polymorphisms with ≥2% missingness were excluded. Genetic data were then imputed to the 1000 Genomes Phase 3 (v5) multi-ethnic reference panel.

Principal component analysis of the genetic data was calculated to evaluate global ancestry, potential batch effects, and residual relatedness among directly genotyped single nucleotide polymorphisms that passed quality control thresholds on both platforms. In prior analyses of the EPOCH cohort [16], associations between a set of 5 single nucleotide polymorphisms (*PNPLA3 rs738409*, *GCKR rs1260326*, *PPP1R3B rs4240624*, *LYPLAL1 rs12137855*, and *NCAN rs2228603*) and adolescent hepatic fat were examined, with only the *PNPLA3* risk allele demonstrating a significant association with hepatic fat content. Therefore, the present analysis focuses exclusively on the *PNPLA3 rs738409* risk allele as a potential effect modifier in the relationship between dietary patterns and adolescent hepatic fat.

### 2.5. Covariates

Maternal pre-pregnancy body mass index (BMI; kg/m^2^) was derived from measured height and pre-pregnancy weight obtained from the medical record. As part of routine prenatal care, all women are routinely screened for gestational diabetes mellitus (GDM) at 24 to 28 weeks using the standard two-step protocol [9,18]. Mothers reported their education level and smoking habits during pregnancy via a questionnaire at study enrollment when the youth were ~9 years of age [19].

Children’s weight was measured on a digital scale and height was measured via a calibrated stadiometer at both research visits. BMI was calculated and standardized using the World Health Organization growth reference [20]. Youth reported their race/ethnicity at enrollment.

Pubertal development was measured at the adolescent study visit, based on Tanner stage of pubic hair development in boys and breast development in girls [21]. Physical activity was assessed at the adolescent study visit using the 3-Day Physical Activity Recall Questionnaire (3DPAR), which captures typical physical activity patterns over a three-day period [22] and has been validated against accelerometry [9,23]. Using 3DPAR, average energy expenditure was derived as a proxy for physical activity (mean metabolic equivalents [METs]/day over a 3-day period).

## 3. Data Analysis

### 3.1. Exploratory Analysis

We began by examining the univariate distributions of variables both for the overall study sample and stratified by *PNPLA3 rs738409* genotype: CC—homozygous for the cytosine variant, which is considered the wild-type or normal/low-risk variant; CG—heterozygous, carrying one cytosine variant and one guanine variant, or intermediate-risk; GG—homozygous for the guanine variant, also known as the “risk” variant.

Next, we examined bivariate relationships between background characteristics and HFF. This step, combined with our prior knowledge of determinants of cardiometabolic health in youth, guided the selection of covariates for the multivariable analysis.

### 3.2. Creation of Dietary Patterns

Using data on dietary intake of 42 food groups, we created dietary patterns using principal component analysis (PCA) as previously described [9]. From the PCA results, we retained the first two factors based on standard criteria, including the Scree plot, eigenvalues > 1 [24], and interpretability [9]. Food groups with factor loadings ≥ |0.20| were considered to be meaningful contributors to a dietary pattern, per convention [25,26].

Using the same set of food groups, we then applied reduced-rank regression (RRR) and focused on the first factor as the dietary pattern, as this is the factor that most strongly predicts the response variable [27]. We then identified overlapping food groups between PCA factors and RRR Factor 1 based on factor loadings.

To derive the integrated PCA-RRR diet score, which represents an eating pattern that is both naturally occurring in this population and associated with HFF, we used RRR factor loadings to weight relevant PCA-identified food groups and took the average across the values. This score is normally distributed (mean ~ 0, SD ~ 1) and can be interpreted the same way dietary patterns are interpreted—i.e., a higher positive score indicates greater alignment of a participant’s eating patterns that are captured by the diet score, whereas a lower and negative score indicates the opposite.

### 3.3. Multivariable Analysis

In the main analysis, we examined associations of the PCA-RRR diet score with HFF using a series of multivariable linear regression models that sequentially adjusted for covariates based on temporality, prior knowledge, and the bivariate analysis. Model 1 included the child’s sex and age, which are standard covariates in all studies of child health outcomes given convention in studies of children’s health, and the centrality of these two variables in accounting for variation in both health behaviors and metabolic outcomes. Model 2 further accounted for perinatal determinants of offspring health, including in utero exposure to maternal GDM, maternal education level, and prenatal smoking habits. Finally, Model 3 accounted for Model 1 covariates plus child BMI z-score and Tanner stage. The rationale behind sequentially adjusting for covariates, as opposed to adjusting for them all at once, is to provide transparency in how the estimate of interest (i.e., the estimate for diet score in relation to HFF) changes across the models. Estimates that are stable in terms of direction, magnitude, and precision of effects provide support for true associations, as opposed to spurious results.

We ran all models using logistic regression to investigate associations of the diet score with elevated liver fat (HFF ≥ 5%) as a dichotomous variable.

In sensitivity analyses, we further adjusted for energy expenditure (METs), which correlates with dietary intake and is associated with metabolic health; as well as self-reported race/ethnicity, which captures sociocultural norms that may shape dietary intake and metabolic disease risk in Model 2.

### 3.4. PNPLA3 rs738409 Genotype

To assess effect modification by the *PNPLA3 rs738409* genotype, we tested for an interaction between the number of copies of the G allele (0, 1, 2) and dietary pattern using Model 1 and subsequently conducted analysis stratified by genotype.

We carried out all analyses using SAS software (version 9.4; SAS Institute Inc., Cary, NC, USA). Across all models, we used alpha = 0.05 as the threshold for statistical significance, though we focused on the direction, magnitude, and precision of associations when interpreting results.

## 4. Results

Table 1 shows the study participant characteristics. At the adolescent visit, the average age was 16.7 ± 1.2 years (range: 12.6 to 19.6 years). Half of the participants were female (50.4%). Half (52.0%) of participants identified as non-Hispanic White, 36.0% as Hispanic, 7.4% as non-Hispanic Black, and 4.7% as non-Hispanic Other. More than half of participants (67.2%) were of normal weight. The average HFF was 2.5% (median: 1.9%; range: 0% to 38.2%) and 7.6% (n = 29) had elevated liver fat consistent with MASLD.

In Supplemental Table S1, we show maternal perinatal and child characteristics among 330 mother–child pairs in the EPOCH study, stratified by *PNPLA3 rs738409* genotype variant. As expected, both BMI and HFF are highest in the GG variant group, though the differences are statistically significant only for HFF. Participants with the high-risk variant (GG allele) were also most likely to be of Hispanic ethnicity.

Table 2 shows bivariate associations of the background characteristics with HFF. Higher maternal pre-pregnancy BMI (*P*-for-trend = 0.0001), lower annual household income (*P*-difference = 0.02), higher BMI z-score at the adolescent study visit (*P*-for-trend < 0.0001), and more advanced Tanner stage were each associated with higher HFF at median age 16 years.

Table 3 shows the composition of dietary patterns we retained from the PCA and the first factor of RRR. In the PCA, we retained two factors. As previously identified in a slightly different subset of this cohort, Factor 1 captured a prudent dietary pattern composed of leafy greens, vegetables, fruit, cruciferous vegetables, and nuts and seeds; and Factor 2 is a Western dietary pattern, composed of fried potatoes, ketchup, beef, and cereal.

Factor 1 from the RRR was driven primarily by Mediterranean-diet-like foods such as vegetables, fruit, nuts and seeds, but also included sports bars, crackers and sandwiches, and beef. Together, we interpret this pattern as a relatively healthy and “active/on-the-go” dietary pattern. The weighted diet score comprised the following food groups from PCA factor 1: vegetables, fruit, nuts and seeds, oatmeal, sports bars, crackers and sandwiches, and beef.

Table 4 shows the association of the PCA-RRR diet score with HFF in the overall study sample. This dietary pattern was inversely associated with both continuous HFF and the odds of elevated HFF, consistent with MASLD. After adjusting for the participants’ age and sex, each 1-unit increment of the diet score corresponded with a 0.48% lower HFF (95% CI: −0.80, −0.16; Model 1). Additional adjustment for perinatal characteristics in Model 2 did not change the estimate. However, adjusting for the participants’ concurrent BMI z-score and Tanner stage in Model 3 attenuated the estimate by ~20%, as expected given that these variables are potential mediators on the causal pathway between diet and HFF, though the estimate remained statistically significant. Similarly, each 1-unit increment of the diet score was associated with 0.46 times the odds of elevated HFF, indicative of MASLD (95% CI: 0.27, 0.78; Model 1), with little change in estimates across subsequent multivariable models.

Table 5 shows results of the analysis stratified by *PNPLA3 rs738409* genotype. In unadjusted models within strata of GG (high-risk), CG (intermediate-risk), and CC (wild-type; low-risk), the inverse association of the diet score with HFF was greatest among participants with the high risk genotype (β: −2.19, 95% CI: −4.35, −0.03), followed by the intermediate (β: −0.43, 95% CI: −0.77, −0.10). We noted weak and null association in the low-risk group. Covariate adjustment attenuated some estimates to the null, which is, in part, an artifact of smaller sample size as the patterns and trends remained apparent. We observed similar associations of the strongest protective effects among individuals with the highest genetic risk when assessing HFF as a binary variable, in accordance with the threshold indicative of MASLD.

In all models, neither adjustment for total energy expenditure (METs) nor adjustment for race/ethnicity changed the findings.

## 5. Discussion

In this study, we applied both unsupervised (PCA) and supervised (RRR) dimension-reduction techniques to dietary data to characterize a dietary pattern that is both naturally occurring and associated with HFF among general-risk youth in the EPOCH study (n = 381). The dietary pattern—which we posit captures an active/on-the-go lifestyle—comprised vegetables, fruit, nuts and seeds, oatmeal, sports bars, crackers and sandwiches, and beef and was inversely associated with HFF and odds of elevated HFF indicative of MASLD. Further, the inverse association of this dietary pattern was most prominent among individuals with the high-risk *PNPLA3 rs738409* variant (GG), suggesting specific dietary modifications and habits that may be particularly beneficial among individuals who are genetically predisposed to fatty liver disease.

### 5.1. Associations of the Dietary Pattern with HFF

The top-loading foods in the PCA-RRR dietary pattern included vegetables, fruit, nuts and seeds, which are key components of prudent and Mediterranean-style diets that are consistently associated with lower HFF in diverse populations of youth [28,29,30] and adults [31], and with results of a 2021 recent systematic review and meta-analysis [32]. To include a few examples from the literature, in a study of 243 Italian adolescents with obesity, poor adherence to a Mediterranean diet was associated with severity of non-alcoholic fatty liver disease [28]. In a study among 181 Turkish children with obesity both with and without fatty liver disease, as well as healthy controls, those with fatty liver disease had the lowest compliance with a Mediterranean-style diet, followed by children with obesity without fatty liver disease, followed by healthy children who demonstrated the highest adherence to a Mediterranean diet [29]. Similarly, in a cross-sectional study of 175 Latino youth aged 8–18 years who were overweight, those who consumed the most non-starchy vegetables had 44% less liver fat than those who consumed the least non-starchy vegetables and those who consumed nutrient-rich vegetables had 17% less visceral adipose tissue compared to those who did not consume nutrient-rich vegetables [30].

Of note, the dietary pattern that we identified in this study includes foods that do not typically align with a prudent or Mediterranean-style dietary pattern, such as sports bars and crackers and sandwiches, some of which are classified as ultra-processed foods. While processed foods have been linked to negative health outcomes, including poor metabolic health [33,34,35,36], some such foods—i.e., sports bars or other packaged foods marketed as being energy- or macronutrient-dense—may reflect an active lifestyle that takes advantage of packaged foods, in moderation, for their convenient and energy-dense nature, to meet higher nutritional demands and they may confer some degree of protection against metabolic conditions like MASLD [37,38], whether directly or via higher physical activity levels. While this interpretation of this dietary pattern requires further exploration and confirmation via both qualitative and quantitative analyses, it aligns with some published findings. For example, a cross-sectional study of 1438 adolescents from public schools in Brazil found that adherence to a “snacking” dietary pattern, which included processed meats, breads, crackers, and cheese, was observed among more physically active individuals [39]. Additionally, a qualitative study among Division I college athletes (n = 14, 64% female) from a variety of sports found that athletes reported commonly consuming sports bars due to them being convenient and quick fuel [40]. Additionally, common ingredients in sports bars such as protein may offer benefits for prevention and management of such diseases [41]. Ultimately, given the unfavorable long-term consequences of regular ultra-processed food consumption, additional research is needed and our findings should be interpreted cautiously.

### 5.2. Effect Modification by PNPLA3 rs738409 Genotype

We found evidence of effect modification by the *PNPLA3 rs738409* genotype. Specifically, the strongest inverse association with dietary pattern was observed in participants with two copies of the risk (G) allele. Such findings suggest that healthful dietary and/or lifestyle modifications may be particularly beneficial for those genetically predisposed to fatty liver disease. In alignment with other multi-ethnic studies [42,43,44,45], EPOCH participants with the high-risk variant of *PNPLA3 rs738409* were also most likely to be of Hispanic ethnicity, thereby driving associations between diet and hepatic fat. As this co-occurrence reflects the true population distribution of the *PNPLA3 rs738409* genotype by race/ethnicity, our findings serve as a foundation for future studies to further investigate specific approaches to preventing MASLD among Hispanic youth.

These findings align with those from a study of 114 Latino adolescents, which observed that the relationship of dietary sugars with liver stiffness, a subclinical indicator of MASLD risk, was strongest among participants with two copies of the risk (G) allele [45]. On the other hand, a recent randomized controlled trial among 105 Latino youth with obesity found that a clinical intervention to reduce dietary sugar did not improve liver outcomes, regardless of *PNPLA3 rs738409* genotype [46]. However, this study only focused on one component of diet (sugary beverages) as opposed to a comprehensive assessment of overall diet, which may explain why the results were null. Further research should aim to better understand the role of the *PNPLA3 rs738409* genotype in the relationships between more comprehensive aspects of diet quality and liver outcomes in youth and adolescents, with and without obesity.

### 5.3. Strengths and Limitations

Our study had several strengths. First, the comprehensive data on confounders and precision covariates substantially improved our ability to make inferences regarding the potential influence of diet on liver fat content. Second, we derived a diet score that contains foods that are both contextually relevant (via PCA) and relevant to HFF (via RRR), which allowed us to identify realistic eating patterns that most strongly predict HFF. Lastly, we considered the role of an established genetic variant (*PNPLA3 rs738409*) associated with obesity-related diseases including fatty liver disease as an effect modifier, which allowed us to better understand heterogeneity in the pathophysiology of fatty liver disease development.

The limitations of this study include the potential for recall bias in dietary intake and residual confounding by environmental and lifestyle characteristics. Further, this study did not use the gold standard of a liver biopsy to measure liver fat content, which limits clinically relevant disease staging and interpretation of severity. However, the EPOCH study used MRI to measure liver fat, which is the most sensitive non-invasive procedure available. The cross-sectional study design of this analysis limits causal inference between diet and hepatic fat, though given that the majority of participants did not meet clinical thresholds of HFF consistent with MASLD and the general-risk nature of this population, it is unlikely that HFF levels would have led to changes to dietary habits. The relatively small sample size, especially within strata of *PNPLA3* polymorphisms, for which only 30 individuals had the high-risk genotype, may limit the statistical power to detect associations, reduce the generalizability of the findings (especially given that EPOCH participants were recruited only from the state of Colorado), and increase susceptibility to selection bias.

## 6. Conclusions

In this analysis of 381 diverse adolescents, compliance with a diet comprising vegetables, fruit, nuts and seeds, oatmeal, sports bars, crackers and sandwiches, and beef was associated with lower HFF and lower odds of elevated HFF currently used as part of the diagnostic criteria for MASLD, especially among youth with a high-risk genotype. While we cannot state with certainty that the “protective” effect of the dietary pattern on the risk of fatty liver disease is causal or reflective of other health behaviors, our findings reinforce the potential utility of strategies to promote healthy lifestyles to interrupt disease progression at early, modifiable phases of the liver disease continuum and the particular benefit of doing so among individuals with a genetic predisposition for metabolic disease. Further, as cardiometabolic disease risk factors track from youth into adulthood [47], our findings support the potential importance of childhood and adolescence as important life stages to mount preventive action, especially among high-risk individuals who may particularly benefit from healthy lifestyle modifications.

## Figures and Tables

**Table 1:** % (N) for maternal perinatal and child characteristics among 381 mother–child pairs in the EPOCH study.

	% (N) ^a^
Maternal perinatal characteristics	
Maternal pre-pregnancy body mass index (BMI; kg/m^2^)	
Underweight (<18.5 kg/m^2^)	3.4% (9)
Normal weight (18.5–24.9 kg/m^2^)	48.3% (130)
Overweight (25.0–29.9 kg/m^2^)	27.5% (74)
Obese (>30 kg/m^2^)	20.8% (56)
Maternal gestational diabetes mellitus	
Yes	17.4% (66)
No	82.6% (314)
Annual household income	
<$25,000	8.2% (31)
$25,000–$49,999	35.8% (136)
>$50,000	56.1% (213)
Maternal education	
<High school	3.2% (12)
High school or equivalent	14.4% (55)
>High school	82.4% (314)
Mother smoked during pregnancy	
Yes	7.4% (28)
No	92.7% (28)
Offspring sex	
Female	50.4% (192)
Male	49.6% (189)
Offspring race/ethnicity	
Non-Hispanic White	52.0% (198)
Hispanic	36.0% (137)
Non-Hispanic Black	7.4% (28)
Non-Hispanic Other	4.7% (18)
Offspring characteristics at the adolescent visit	
Age	
12 to <16 years	26.8% (102)
16 to <17 years	29.1% (111)
≥17 years	44.1% (168)
BMI z-score ^b^	
<−2.0	2.1% (8)
≥−2.0 to ≤1.0	67.2% (256)
>1.0 to ≤2.0	23.1% (88)
>2.0	7.6% (29)
Pubertal status ^c^	
Tanner stage 1	0.0% (0)
Tanner stage 2	0.8% (3)
Tanner stage 3	5.0% (19)
Tanner stage 4 or 5	94.2% (359)

^a^ Totals may not add up to 381 due to missing values. ^b^ According to the World Health Organization (WHO) growth reference for children 5 to 19 years of age. ^c^ Based on pubic hair development in boys and breast development in girls.

**Table 2:** Bivariate associations of background characteristics with hepatic fat fraction among 381 mother–child pairs in the EPOCH study.

	N ^a^	Hepatic Fat Fraction	*p* ^b^
	381	2.47 ± 3.11	
		Range: 0–38.21	
Maternal perinatal characteristics			
Maternal pre-pregnancy body mass index (BMI; kg/m^2^)			**0.0001**
Underweight (<18.5 kg/m^2^)	9	2.19 ± 0.90	
Normal weight (18.5–24.9 kg/m^2^)	130	1.89 ± 1.17	
Overweight (25.0–29.9 kg/m^2^)	74	2.56 ± 2.20	
Obese (>30 kg/m^2^)	56	3.78 ± 5.69	
Maternal gestational diabetes mellitus			0.34
Yes	66	2.80 ± 4.72	
No	314	2.40 ± 2.67	
Annual household income			**0.02**
<$25,000	31	3.74 ± 5.57	
$25,000–$49,999	136	2.54 ± 3.52	
>$50,000	213	2.24 ± 2.19	
Maternal education			0.87
<High school	12	2.60 ± 2.18	
High school or equivalent	55	2.50 ± 2.33	
>High school	314	2.46 ± 3.27	
Mother smoked during pregnancy			0.54
Yes	28	2.81 ± 5.59	
No	353	2.44 ± 2.84	
Offpsing sex			0.16
Female	192	2.24 ± 2.22	
Male	189	2.70 ± 3.81	
Offspring race/ethnicity			0.18
Non-Hispanic White	198	2.00 ± 1.33	
Hispanic	137	3.27 ± 4.80	
Non-Hispanic Black	28	2.26 ± 1.31	
Non-Hispanic Other	18	1.89 ± 0.93	
Offspring characteristics at the adolescent visit			
Age			0.23
12 to <16 years	102	3.06 ± 4.02	
16 to <17 years	111	1.92 ± 1.37	
≥17 years	168	2.47 ± 3.26	
BMI z-score ^c^			**<0.0001**
<−2.0	8	1.93 ± 1.10	
≥−2.0 to ≤1.0	256	1.79 ± 1.01	
>1.0 to ≤2.0	88	3.16 ± 4.35	
>2.0	29	6.48 ± 6.39	
Pubertal status ^d^			**0.0001**
Tanner stage 1	0		
Tanner stage 2	3	14.66 ± 20.40	
Tanner stage 3	19	1.93 ± 0.93	
Tanner stage 4 or 5	359	2.40 ± 2.58	
METs over a 3-day period			0.73
Q1 (lowest)	90	2.68 ± 2.90	
Q2	94	2.35 ± 1.73	
Q3	97	2.48 ± 3.90	
Q4 (highest)	91	2.46 ± 3.62	

^a^ Totals may not add up to 381 due to missing values. ^b^ From a *p* for linear trend for ordinal variables; from a Type 3 test for a difference for categorical variables (child sex only). ^c^ According to the World Health Organization (WHO) growth reference for children 5 to 19 years of age. ^d^ Based on pubic hair development in boys and breast development in girls. Bolded values indicate statistical significance at alpha = 0.05.

**Table 3:** Top-loading food groups from principal component analysis (PCA) and reduced-rank regression (RRR) during the adolescent visit (median age 16 years, range 12–19 years).

Food Group	PCA		RRR
	Factor 1: Prudent	Factor 2: Western	Factor 1: Prudent
Leafy greens	68		
Vegetables	68 *		−27 *
Fruit	58 *		−21 *
Cruciferous vegetables	46		
Nuts and seeds	46 *		−25 *
Yogurt	44		
Vegetable stir fry	40	21	
Granola bars	32		
Oatmeal	32 *		−20 *
Sports bars	29 *		−28 *
Beans	27		
Fish	26		
Crackers and sandwiches	24 *		−32 *
Eggs	23		
Cheese	23		
Potatoes and sweet potatoes	22		
Dried fruit	22		
Veggie or beef soup	20		
High-fat sandwich or wrap	−26	22	
Margarine, butter, or lard	−34		
Sugar-sweetened beverages	−35		
Fried potatoes	−25	58	
Ketchup		52	
Beef	−36 *	44 *	25 *
Fast food	−35	42	
Salad dressing	38	41	
High-fat snacks		36	
Candy	22	33	
Ice cream		30	
Dessert		23	−20
Chicken		23	
Pork		21	
Milk		−33	
Cereal		−45	
Fruit juice			−21
High-fat sides			20

Bolded columns with asterisks indicate the food groups that were included in the diet score.

**Table 4:** Associations (β [95% CI] or OR [95% CI]) of diet score with hepatic fat among 381 youth in the EPOCH study during the adolescent visit (median age 16 years, range 12–19 years).

		Diet Score, per 1 SD	
Model	Model N	Hepatic Fat Fraction (%): β (95% CI)	Elevated Hepatic Fat Fraction Yes/No: OR (95% CI)
Unadjusted	N = 381	**−0.51 (−0.82, −0.20)**	**0.47 (0.28, 0.79)**
Model 1	N = 380	**−0.48 (−0.80, −0.16)**	**0.46 (0.27, 0.78)**
Model 2	N = 379	**−0.48 (−0.81, −0.16)**	**0.46 (0.27, 0.79)**
Model 3	N = 380	**−0.39 (−0.69, −0.10)**	**0.49 (0.28, 0.88)**

Bolded values indicate statistical significance at alpha = 0.05. Model 1: Adjusted for child sex and age. Model 2: Model 1 + in utero exposure to maternal GDM, maternal education level, and prenatal smoking habits. Model 3: Model 1 + child BMI z-score and Tanner stage.

**Table 5:** Associations (β [95% CI] or OR [95% CI]) of diet z-score with hepatic fat stratified by *PNPLA3 rs738409* genotype among 330 youth in the EPOCH study at ages 12–19 years.

	*PNPLA3 rs738409 Genotype*		
	CC (Wild-Type) N = 175	CG (Intermediate) N = 125	GG (High-Risk) N = 30
	Diet z-Score, per 1 SD		
Hepatic fat fraction (%): β (95% CI)			
Unadjusted	−0.32 (−0.77, 0.13)	**−0.43 (−0.77, −0.10)**	**−2.19 (−4.35, −0.03)**
Model 1	−0.23 (−0.69, 0.22)	**−0.44 (−0.81, −0.08)**	−1.76 (−4.10, 0.57)
Model 2	−0.25 (−0.70, 0.20)	**−0.37 (−0.74, −0.00)**	−1.33 (−3.55, 0.88)
Elevated hepatic fat fraction yes/no: OR (95% CI)			
Unadjusted	0.62 (0.26, 1.49)	0.61 (0.28, 1.34)	**0.10 (0.01, 0.84)**
Model 1	0.71 (0.29, 1.70)	0.55 (0.23, 1.29)	**0.09 (0.01, 0.99)**
Model 2	0.70 (0.29, 1.70)	0.68 (0.28, 1.65)	0.05 (0.00, 1.03)

Bolded values indicate statistical significance at alpha = 0.05. Model 1: Adjusted for child sex and age. Model 2: Model 1 + in utero exposure to maternal GDM, maternal education level, and prenatal smoking habits.

## Data Availability

The data presented in this study are available on request from the corresponding author due to data restrictions.

## Supplementary Materials

The following supporting information can be downloaded at https://www.mdpi.com/article/10.3390/nu18071087/s1, Table S1: % (N) for maternal perinatal and child characteristics among 330 mother-child pairs in the EPOCH study, stratified by *PNPLA3 rs738409* Genotype variant.

## Abbreviations

The following abbreviations are used in this manuscript:

HFF	Hepatic Fat Fraction
MRI	Magnetic Resonance Imaging
MASLD	Metabolic Dysfunction-Associated Steatotic Liver Disease
EPOCH	Exploring Perinatal Outcomes Among CHildren
PCA	Principal Component Analysis
RRR	Reduced-Rank Regression
FFQ	Food Frequency Questionnaire
GDM	Gestational Diabetes Mellitus
3DPAR	3-Day Physical Activity Recall Questionnaire
METs	Mean Metabolic Equivalents
BMI	Body Mass Index
