# Supplementary material for: Dietary Patterns, Hepatic Fat Fraction, and the Role of Genotype
Source: Nutrients. 2026 Mar 28;18(7):1087. doi: 10.3390/nu18071087 (PMC13074321; doi:10.3390/nu18071087)
Supplement: Supplementary file 1 [file nutrients-18-01087-s001.zip › nutrients-4153912-supplementary.pdf]

Supplemental Table S1 % (N) for maternal perinatal and child characteristics among 330 mother-child pairs in the EPOCH study, stratified by *PNPLA3 rs738409* Genotype variant.

	Mean ± SD or % (N)[a]			
	CC (wild type) N=175	***CG (intermediate) N=125***	***GG (high-risk) N=30***	***P***[b]
Maternal perinatal characteristics				
Maternal pre-pregnancy body mass index (BMI; kg/m^2)				0.07
Underweight (<18.5 kg/m^2)	1.6% (2)	3.5% (3)	15.4% (4)	
Normal weight (18.5-24.9 kg/m^2)	53.2% (66)	49.4% (42)	42.3% (11)	
Overweight (25.0-29.9 kg/m^2)	26.6% (33)	25.9% (22)	23.1% (6)	
Obese (>30 kg/m^2)	18.6% (23)	21.2% (18)	19.2% (5)	
Maternal gestational diabetes mellitus				0.20
Yes	20.7% (36)	14.4% (18)	10.0% (3)	
No	79.3% (138)	85.6$ (107)	90.0% (27)	
Annual household income				**0.04**
<$25,000	6.9% (12)	4.8% (4)	20.0% (6)	
$25,000-$49,999	31.4% (55)	39.5% (49)	33.3% (10)	
>$50,000	61.7% (108)	55.7% (69)	46.7% (14)	
Maternal education				0.14
<High school	4.0% (7)	1.6% (2)	3.3% (1)	
High school or equivalent	10.9% (19)	13.6% (17)	26.7% (8)	
>High school	85.1% (149)	84.8% (106)	70.0% (21)	
Mother smoked during pregnancy				0.12
Yes	7.4% (13)	5.6% (7)	16.7% (5)	
No	92.6% (162)	84.4% (118)	83.3% (25)	
Offspring sex				0.38
Female	47.4% (83)	55.2% (69)	46.7% (14)	
Male	52.6% (92)	44.8% (56)	53.3% (16)	
Offspring race/ethnicity				**<0.0001**
Non-Hispanic White	61.1% (107)	54.4% (68)	26.7% (8)	
Hispanic	23.4% (41)	40.0% (50)	70.0% (21)	
Non-Hispanic Black	9.7% (17)	4.0% (5)	0.0% (0)	
Non-Hispanic Other	5.7% (10)	1.6% (2)	3.3% (1)	
Offspring characteristics at the adolescent visit				
Age				0.41
12 to <16 years	28.0% (49)	24.0% (30)	33.3% (10)	
16 to <17 years	29.7% (52)	42.0% (30)	30.0% (9)	
≥17 years	42.3% (74)	52.0% (65)	36.7% (11)	
BMI z-score[c]				0.17
< -2.0	0.6% (1)	3.2% (4)	3.3% (1)	
≥ -2.0 to ≤1.0	69.1% (121)	70.4% (88)	66.7% (20)	
>1.0 to ≤2.0	22.9% (40)	21.6% (27)	13.3% (4)	
>2.0	7.4% (13)	4.8% (6)	16.7% (5)	
Pubertal status[d]				0.83
Tanner stage 1	0.0% (0)	0.0% (0)	0.0% (0)	
Tanner stage 2	0.6% (1)	1.6% (2)	0.0% (0)	
Tanner stage 3	4.6% (8)	5.6% (7)	6.7% (2)	
Tanner stage 4 or 5	94.9% (166)	92.8% (116)	93.3% (28)	
Hepatic fat fraction (%)	2.25 ± 3.1	2.37 ± 1.9	3.86 ± 5.5	**0.03**

Bolded values indicate statistical significance at alpha = 0.05.

[a] Totals may not add up to 381 due to missing values.

[b] From a Wald Chi-squared test.

[c] According to the World Health Organization (WHO) growth reference for children 5 to 19 years of age.

[d] Based on pubic hair development in boys and breast development in girls.
